# Enhanced Hardness-Toughness Balance Induced by Adaptive Adjustment of the Matrix Microstructure in In Situ Composites

**DOI:** 10.3390/ma16124437

**Published:** 2023-06-16

**Authors:** Mingjuan Zhao, Xiang Jiang, Yumeng Guan, Haichao Yang, Longzhi Zhao, Dejia Liu, Haitao Jiao, Meng Yu, Yanchuan Tang, Laichang Zhang

**Affiliations:** 1 Key Laboratory of Advanced Materials for Vehicles and Laser Additive Manufacturing of Nanchang City, East China Jiaotong University, Nanchang 330013, China; 2 Key Laboratory of Vehicle and Equipment of Education Ministry, East China Jiaotong University, Nanchang 330013, China; 3 State Key Laboratory of Performance Monitoring and Protecting of Rail Transit Infrastructure, East China Jiaotong University, Nanchang 330013, China; 4 Nanchang Railway Tongda Industry and Trade Co., Ltd., Nanchang 330026, China; 5 School of Engineering, Edith Cowan University, 270 Joondalup Drive, Joondalup, Perth, WA 6027, Australia

**Keywords:** in situ bainite steel matrix composite, direct laser deposition, adaptive adjustment of matrix microstructure, good hardness-toughness balance

## Abstract

With the development of high-speed and heavy-haul railway transportation, the surface failure of rail turnouts has become increasingly severe due to insufficient high hardness-toughness combination. In this work, in situ bainite steel matrix composites with WC primary reinforcement were fabricated via direct laser deposition (DLD). With the increased primary reinforcement content, the adaptive adjustments of the matrix microstructure and in situ reinforcement were obtained at the same time. Furthermore, the dependence of the adaptive adjustment of the composite microstructure on the composites’ balance of hardness and impact toughness was evaluated. During DLD, the laser induces an interaction among the primary composite powders, which leads to obvious changes in the phase composition and morphology of the composites. With the increased WC primary reinforcement content, the dominant sheaves of the lath-like bainite and the few island-like retained austenite are changed into needle-like lower bainite and plenty of block-like retained austenite in the matrix, and the final reinforcement of Fe_3_W_3_C and WC is obtained. In addition, with the increased primary reinforcement content, the microhardness of the bainite steel matrix composites increases remarkably, but the impact toughness decreases. However, compared with conventional metal matrix composites, the in situ bainite steel matrix composites manufactured via DLD possess a much better hardness-toughness balance, which can be attributed to the adaptive adjustment of the matrix microstructure. This work provides a new insight into obtaining new materials with a good combination of hardness and toughness.

## 1. Introduction

With the development of high-speed and heavy-haul railway transportation, rolling-contact fatigue crack and peeling on the surface of rail turnouts have become increasingly severe [1,2,3]. The surface failure of rail turnouts severely reduces their service life, which leads to increased operation costs and potential safety hazards [4]. It is well known that the failure of rail turnouts is closely related to the insufficient hardness and toughness of the components [5]. Hence, it is of great importance to develop a rail turnout material with high hardness and high toughness.

Compared with the conventional surface treatment methods (such as thermal spraying, plasma spraying), laser surface-treatment technologies demonstrate obvious advantages, such as a small heat effect zone, good interface bonding, high reliability and high precision [6,7]. Specifically, during direct laser deposition (DLD), a high-energy laser beam is used to melt the composite powder, and then the melts are deposited on the substrate surface to form the bulk deposition. During the DLD process, the composition and microstructure of the deposition can be regulated [8], which makes the DLD technology suitable for strengthening the surface of the materials. For example, martensite Fe-based alloys with good wear resistance can be deposited on the surface of rail components via DLD [9,10,11,12,13,14,15]. However, martensite Fe-based alloys often demonstrate insufficient toughness [9], and cannot meet the demand of the rail component suffering impact load. Fe-based alloys with a bainite microstructure have a good balance of wear resistance and toughness; therefore, isothermal heat treatment is used for Fe-based alloys in order to change their martensite microstructure into bainite, but the heat treatment needs a long time, which cannot meet the low production cycle of the rail component. Furthermore, the wear resistance of bainite steel is not high enough to suit rail turnouts [13,15].

It has been reported that metal matrix composites (MMCs) exhibit excellent mechanical properties, such as a high modulus, high strength and good wear resistance, compared with their primary alloy matrix [16,17,18]. Thus, MMCs with a high volume fraction reinforcement are required in order to enhance the wear resistance of rail turnouts [19,20]. However, the toughness of MMCs with high volume fraction reinforcement is low [21,22,23]. Consequently, it is difficult to obtain a balance between high toughness and good wear resistance in conventional MMCs [24,25,26,27]. 

In conventional MMCs manufactured via powder metallurgy, their casting, matrix microstructure and composition are almost not changed compared with their primary alloy matrix. Therefore, the enhancement of the composites’ performance is mainly attributed to the strengthening of the reinforcement, but has nothing to do with the matrix. However, during laser deposition, the complex interaction between the deposited composite powder irradiated by the high-energy-density laser induces the in situ action and the solution in the deposited powder, thus making the matrix constituent and microstructure change with the increased primary reinforcement volume fraction.

Al Mangour [28,29,30,31] suggested that that the particle reinforcement with relatively small size would melt during manufacturing TiC reinforced 316L stainless steel matrix composites by selective laser melting(SLM). Furthermore, the content of the ferrite phase (α-Fe) in the matrix increases with the increased TiC reinforcement content. The melting of the particle reinforcement, irradiated by a high-energy laser beam, has also been widely reported in the literature [32,33,34]. This phenomenon leads to a large deviation in the microstructure and properties of the composites without the interaction between the primary matrix and reinforcement.

Accordingly, the interaction between the primary matrix and reinforcement can also be utilized to regulate the microstructure of the composite matrix. In our previous work, a bainite steel matrix composite was fabricated, and the decomposition or dissolution of the reinforcing particles in the matrices were also found due to the extremely high temperature during the DLD process, which changes the chemical constituent and microstructure of the matrix [35,36,37]. Furthermore, owing to the change in the matrix constituent, the content of retained austenite in the matrix was found to vary in a large range spontaneously. Therefore, the adaptive change in the matrix comes from the content of primary particle reinforcement, which provides a much more convenient way to regulate the toughness of the composites. 

Due to the combination of a high hardness and low thermal expansion coefficient, WC particle reinforcement is often used in Fe-based composites [38]. In addition, the dissolution of the W and C elements derived from WC particles exert a significant influence on the transformation of undercooled austenite, which can be used to regulate the microstructure of the Fe-based alloy matrix [39]. In this work, WC particles were used as the primary reinforcement, and the effect of the volume fraction of primary reinforcement on the matrix microstructure and mechanical properties of the in situ bainite steel matrix composite was investigated. Finally, the dependence of the adaptive adjustment of the matrix on the composites’ balance of hardness and impact toughness was evaluated. This work provides new insights into obtaining new materials with a good combination of hardness and toughness.

## 2. Materials and Methods

The bainite steel matrix composite was fabricated via DLD on a U75V steel substrate (a kind of railway material). The surface of the substrate was ground and then sand blasted in order to remove the surface oxide layer. The gas-atomized Fe-based alloyed powder with a particle size of 50~70 μm in diameter was applied to construct the matrix of the composite. The chemical compositions (in wt%) of the substrate and Fe-based alloyed powder are shown in Table 1. In order to avoid the sputtering of the tungsten carbide ceramic particle from the composite powder by the laser during laser deposition, tungsten carbide coated with a Co layer was used as a primary reinforcement. The composite powder containing the Fe-based powder and WC powder were thoroughly mixed using a planetary ball mill in an argon atmosphere at a speed of 200 rpm for 2 h. Finally, the composite powders were dried in a vacuum furnace at 80 °C for 2 h.

**Figure 1:**
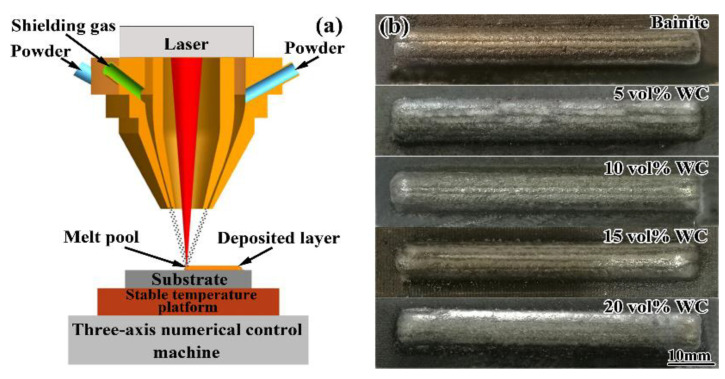
(**a**) Schematic of the laser processing system and (**b**) macroscopic morphology of the as-built composites.

The bainite steel matrix composites were manufactured using a laser processing system (as shown in Figure 1) comprising a semiconductor laser device with a maximum output power of 2.5 kW (LDM-2500-60, Laserline, Mülheim-Kärlich, Germany), a three-axis numerical control machine controlling the laser scanning path, a powder coaxial nozzle feeding system with a shielding gas device and a stable temperature platform. The process involved the following 3 steps. Firstly, the substrates were heated to 300 ± 5 °C in the resistance furnace using argon protection and then placed on a platform with the pre-set heated temperature of 300 °C to avoid martensite transformation during laser deposition. Afterwards, the composite powder was deposited on the surface of the substrates using DLD technology. The processing parameters were as follows: laser power of 800 W, laser spot diameter of 1.5 ± 0.1 mm, overlap ratio of 40% and scanning velocity of 360 mm/min. As shown in Figure 1b, the samples had a good surface quality and no macroscopic cracks were observed. Finally, the composite samples were put into a 300 ± 3 °C salt bath for isothermal treatment for 200 min, and the final composites, air cooled to room temperature (RT), were obtained. Specimens were sectioned using electric discharge wire cutting to obtain the composite samples and characterize their microstructure and mechanical properties.

An optical microscope (OM, Carl Zeiss Jena Axio Vert.A1) and a field-emission scanning electron microscope (FESEM, Nova Nano SEM450) were used to characterize the microstructural features. A backscattered electron (BSE) mode of FESEM was used to distinguish the reinforcements and steel matrix. The composition of the samples was analyzed using an energy-dispersive X-ray spectrometer (EDS) equipped on the FESEM. X-ray diffraction (XRD, D8 Advance) analyses with a Cu target were conducted for phase identification.

The microhardness of the samples was measured using a Vickers microhardness tester (Duramin-40, Struers, Denmark), with a 200 g load and a 10 s dwell time. Charpy U-notched impact tests were conducted with 55 mm × 10 mm × 10 mm samples on a pendulum impact machine (PTMS4300, Suns, China) at the RT. The notch was prepared perpendicular to the laser deposition direction. The reported impact toughness of each sample was averaged from three independent tests. The fracture surfaces of the impact samples were observed via FESEM. 

## 3. Results

### 3.1. XRD Analysis

**Figure 2:**
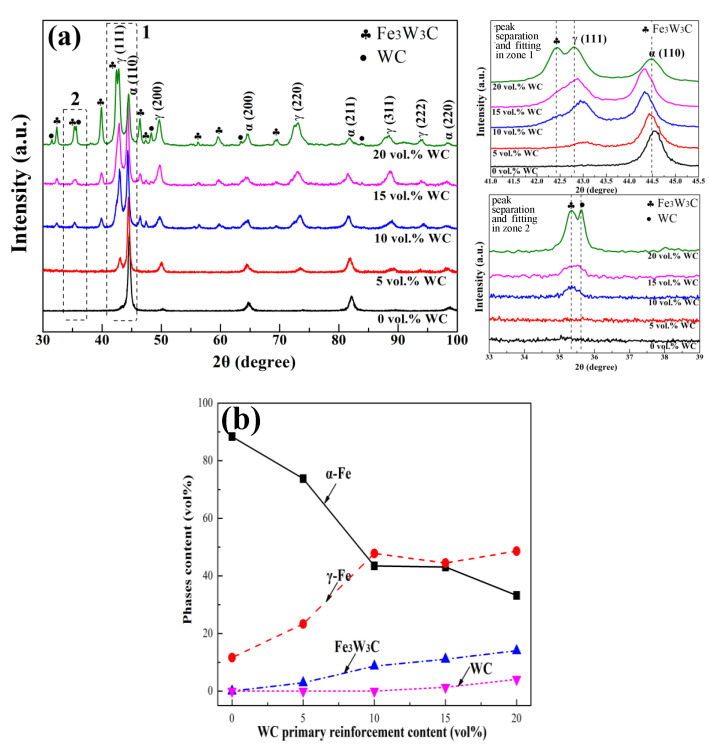
(**a**) XRD patterns and (**b**) the corresponding analyzed phase content in the bainite steel matrix composites.

Figure 2 illustrates the phase constituents and their relative contents in the bainite steel matrix composite. As shown in Figure 2a, the matrix of the composite is changed from a mainly ferrite phase (α-Fe) with a little austenite phase (γ-Fe) to α-Fe with a considerable amount of γ-Fe when WC primary reinforcements are added. Furthermore, a Fe_3_W_3_C phase appears instead of a WC phase when the addition of primary reinforcement is relatively low. The WC phase presents together with the Fe_3_W_3_C phase when the primary reinforcement exceeds 15 vol%. The volume fraction of different phases was further quantified, as shown in Figure 2b. In the case of peak overlapping (the magnified images in Figure 2a), a Pearson VII function was used for the peak separation and fitting [15,40,41,42]. As the volume fraction of the WC primary reinforcement is increased, the volume fraction of the α-Fe phase declines; however, that of the γ-Fe phase increases. The volume fraction of the γ-Fe phase is not higher than that of the α-Fe phase until the WC primary reinforcement content reaches 20 vol%. As for the carbides, the volume fraction of Fe_3_W_3_C increases approximately linearly with the increased primary reinforcement content, and its maximum value is about 14 vol%. However, with the increased WC primary reinforcement content, the final WC content of the composites is maintained at zero when the content is less than 10%; meanwhile, when the content of primary reinforcement is higher than 10 vol%, the volume fraction of the final WC rises. When the content of primary reinforcement is 20%, the final WC reinforcement content in the composites is 4.1 vol%. 

### 3.2. Microstructure

**Figure 3:**
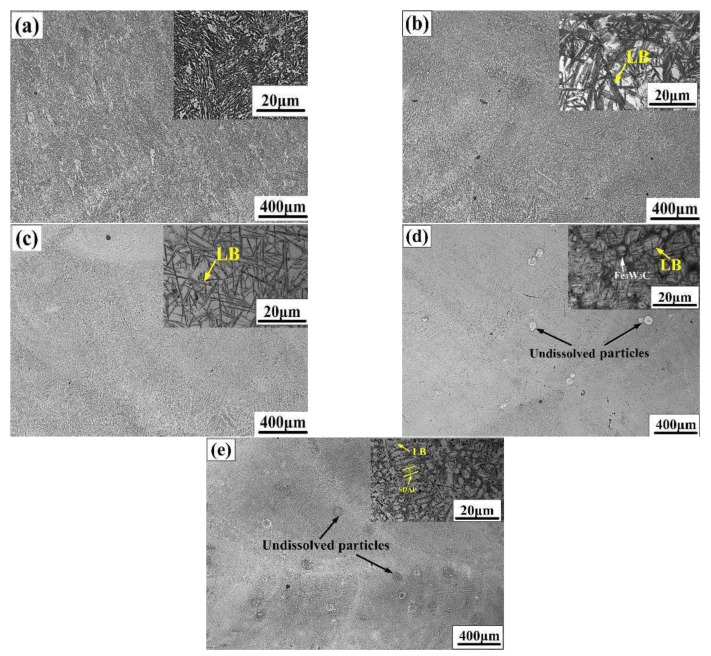
Microstructure evolution of the bainite steel matrix composites with the increased WC primary reinforcement volume fractions of (**a**) 0%, (**b**) 5%, (**c**) 10%, (**d**) 15%, and (**e**) 20%.

Figure 3 presents the optical micrographs of the bainite steel matrix composites with different volume fractions of WC. For the bainite steel without WC, the morphology of prior austenite grains can hardly be recognized. However, the prior austenite grains in the bainite steel matrix composites show a typical dendritic shape. Meanwhile, both the primary dendrite arm spacing (PDAS) and secondary dendrite arm spacing (SDAP) decrease with the increased volume fraction of WC. When the WC primary reinforcement content is higher than 15 vol%, plenty of white undissolved particles can be observed. The average diameter of the particles is about 47 μm, which is a little smaller than the average particle size of WC powder (about 65 μm). With the increased WC volume fraction, the bainite morphology changes from a lath shape to a needle shape and the content of block-like retained austenite (RA) increases, which is consistent with the XRD results (Figure 2b). Furthermore, the black network-like microstructure begins to emerge in the interdendritic region when the WC primary reinforcement volume fraction is higher than 10%.

**Figure 4:**
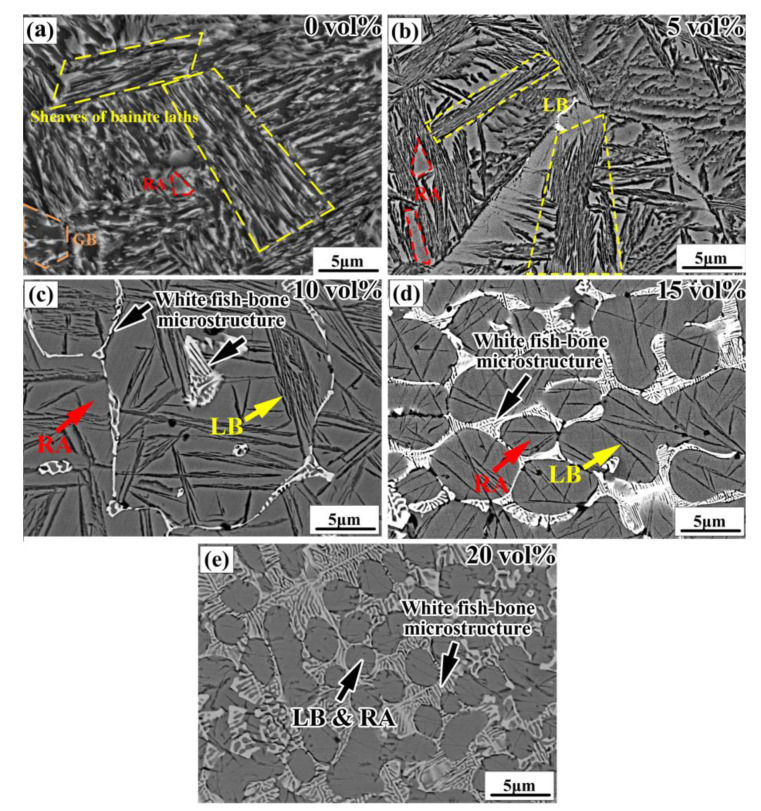
SEM images of the bainite steel matrix composites with the increased volume fraction of WC primary reinforcement (**a**) 0%, (**b**) 5%, (**c**) 10%, (**d**) 15%, and (**e**) 20%.

The fine microstructure of the bainite steel matrix composites was further investigated using the BSE mode of SEM, as shown in Figure 4. As for the bainite steel with no WC addition (Figure 4a), the bainite steel mainly consists of sheaves of lath-like bainite, a few granular bainite (GB) and island-like RA. With the increased WC reinforcement volume fraction, the lath-like bainite and GB transform into black needle-like lower bainite (LB), and the length and width of the LB needles decrease gradually (Figure 4b–e). Meanwhile, the morphology of RA also changes from an island-like to block-like shape. For the bainite steel matrix composite with a relatively high volume fraction of WC (no less than 10 vol%), the white fish-bone-shaped microstructure appears at the boundary of the prior austenite grains (Figure 4d,e). With the increased addition of WC, the area of the intergranular region occupied by the white fish-bone-shaped microstructure increases; at the same time, the prior austenite grain is refined. 

**Figure 5:**
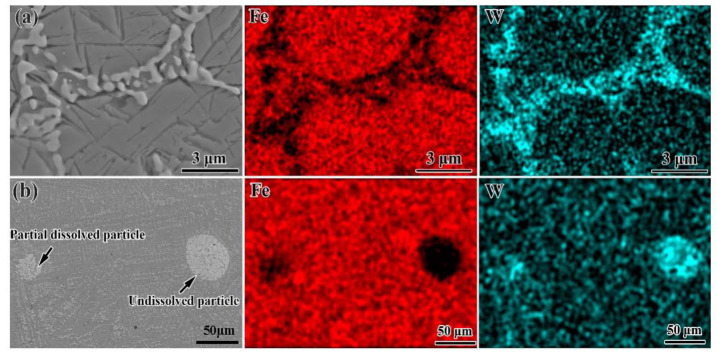
EDS mapping results at different regions: (**a**) intergranular region and (**b**) undissolved particles for the bainite steel matrix composite with 15 vol% WC primary reinforcement.

According to the phase constituent and the microstructure of the bainite steel matrix composite with 15 vol% WC particles, EDS analysis was conducted to further identify the phase composition of the intergranular region and undissolved particles. As shown in Figure 5a, the elemental maps of the intergranular region indicate that W enriches the white fish-bone-shaped microstructure. Combining the volume fraction of the white fish-bone-shaped microstructure obtained from the SEM images with the phase analysis results from the XRD patterns, the white fish-bone-shaped phase is Fe_3_W_3_C. The elemental distributions of the partial dissolved particles and the undissolved particles are presented in Figure 5b. Much W and little Fe can be detected in the partially dissolved WC particles region. Meanwhile, in the undissolved WC particles region, the enrichment degree of W in the interior of the particles is much higher than that of the partially dissolved particles, and no Fe is detected.

### 3.3. Mechanical Properties

**Figure 6:**
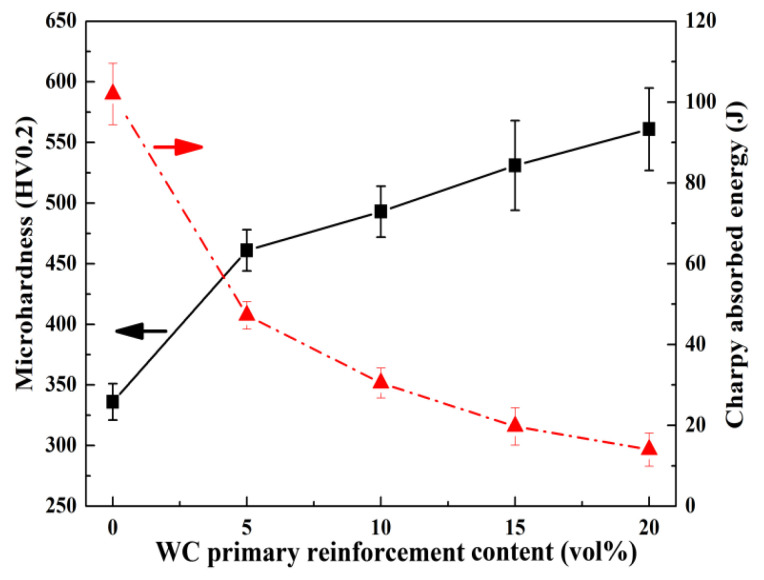
Microhardness and impact toughness of the bainite steel matrix composites.

As shown in Figure 6, the microhardness of the bainite steel is about 330 HV0.2, which is lower than that of the U75V steel substrate (375 HV0.2). The microhardness of the DLD manufactured bainite steel matrix composites is much higher than that of the U75V steel substrate. With the increased primary reinforcement content, the microhardness of bainite steel matrix composites increases remarkably. The microhardness increases rapidly to 461 HV0.2 when the WC primary reinforcement volume fraction is only 5 vol%, which is approximately 40% higher than that of the bainite steel. Moreover, the microhardness of composites with 20 vol% primary reinforcement is increased to 561 HV0.2. In contrast, the impact toughness of the bainite steel matrix composite decreases with the increased volume fraction of WC primary reinforcement (Figure 6). However, the impact toughness of the composite when the primary reinforcement volume fraction is less than 10% is still higher than that of the U75V steel substrate (26 J), which can satisfy the demand of the rail turnout service.

**Figure 7:**
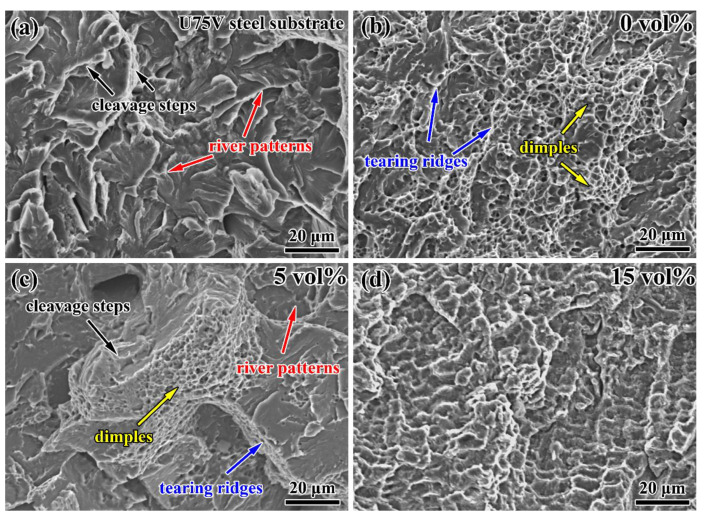
The impact fracture surface morphology of (**a**) the U75V steel substrate and (**b**–**d**) bainite steel matrix composites with different volume fractions of WC primary reinforcement.

Figure 7 indicates the impact fracture surface morphology of the U75V steel substrate and bainite steel matrix composites with different volume fractions of WC primary reinforcement. The U75V steel substrate shows a typical feature of cleavage fracture, which consists of cleavage steps and river patterns (Figure 7a). In contrast, dimples and tearing ridges are observed in the fracture of the bainite steel (Figure 7b), which indicates that the facture mechanism occurring is microvoids coalescence ductile fracture. Figure 7c presents the fracture of the bainite steel matrix composite with 5 vol% primary reinforcement. Both the features of ductile fracture (dimples and tearing ridges) and cleavage fracture (cleavage steps and river patterns) are evident on the fracture surface. This suggests that the fracture mechanism occurring is quasi-cleavage fracture in the composites. The crystal sugar fracture (Figure 7d) indicates that the bainite steel matrix composite with 15 vol% primary reinforcement follows the brittle intergranular fracture mechanism.

## 4. Discussion

### 4.1. Formation Mechanism of the Reinforcement during Laser Deposition

**Figure 8:**
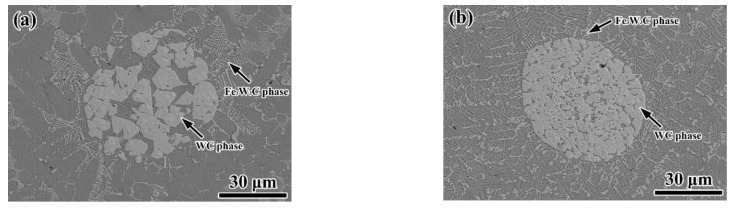
The morphology of the Fe_3_W_3_C adjacent to the (**a**) partially dissolved WC particle and (**b**) undissolved WC particle.

Information about the phases in the bainite steel matrix composite that was revealed by XRD (Figure 2) indicates that the in situ Fe_3_W_3_C reinforcement is the main reinforcement in the composite when the volume fraction of primary reinforcement WC is not higher than 10%. When the primary reinforcement volume fraction increases from 10% to 20%, the final reinforcement in the composites includes Fe_3_W_3_C in situ reinforcement and WC primary reinforcement. Moreover, the content of Fe_3_W_3_C is much higher than that of WC. Therefore, it can be deduced that the decomposed WC during laser deposition has dissolved in the bainite steel matrix and participated in the formation of Fe_3_W_3_C in the matrix. This is proved by the morphology of the Fe_3_W_3_C phase adjacent to the WC particles (Figure 8). The Fe_3_W_3_C phase in the vicinity of the partially dissolved WC particle presents the feature of a continuously fish-bone-shaped microstructure. The content of the Fe_3_W_3_C phase decreases with the increased distance from the partially dissolved WC particles. In contrast, compared with the Fe_3_W_3_C phase near the partially dissolved WC particle, the Fe_3_W_3_C phase near the undissolved WC particle has a much lower content and the carbides are distributed more uniformly.

For the preparation of WC reinforced Fe-based matrix composites, the temperature of the material during direct laser deposition is always higher than that during traditional powder metallurgy and casting process [43,44,45]. Under the irradiation of a laser beam with an extremely high-energy density, the maximum temperature of the metal molten pool can exceed 3000 °C, which is higher than the decomposition temperature of WC (1250 °C) [46]. Then, the WC primary reinforcement will react with the steel matrix. The reaction is as follows [47,48]:

2WC→W_2_C + C (1)

W_2_C→2W + C (2)

WC + W + L(rich in Fe) → Fe_3_W_3_C (M_6_C) (3)

Additionally, the high temperature gradient of the molten pool during the DLD process brings the obvious convection of liquid metal, which is beneficial to a successful reaction [49]. When the addition of WC is low (less than 10 vol% in this work), all WC particles participate in the formation of Fe_3_W_3_C in the bainite steel matrix. In contrast, when WC is excessive, such as the 15 vol% WC in this work, both the Fe_3_W_3_C in situ reinforcement and WC primary reinforcement exist in the matrix at the same time. Free W atoms, which join to form Fe_3_W_3_C, come from the dissolved WC particle. Therefore, the concentration of W atoms around the partially dissolved WC particles is higher than that of the matrix far from the WC particles. Accordingly, the in situ Fe_3_W_3_C content near the partially dissolved WC particles is high.

### 4.2. Mechanism of the Adaptive Adjustment of the Matrix Microstructure with Primary Reinforcement

**Figure 9:**
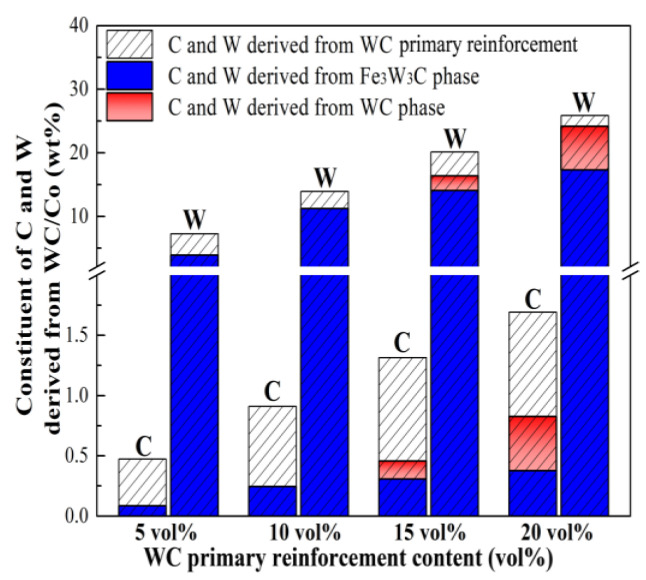
Constituent of C and W derived from primary reinforcement in the composites.

With the increased volume fraction of WC primary reinforcement, not only the constituent and morphology of the final reinforcement in the composites are changed, but also the microstructure of the bainite steel matrix is significantly altered (Figure 3 and Figure 4), which is attributed to the change in the solute W and C content in the bainite steel matrix. Figure 9 presents the constituent of C and W derived from the WC primary reinforcement. It can be concluded that the formation of the Fe_3_W_3_C phase and WC phase does not consume all the C and W derived from the WC primary reinforcement and the remaining C and W dissolve in the bainite steel matrix, which significantly retards the transformation of undercooled austenite [50,51].

**Figure 10:**
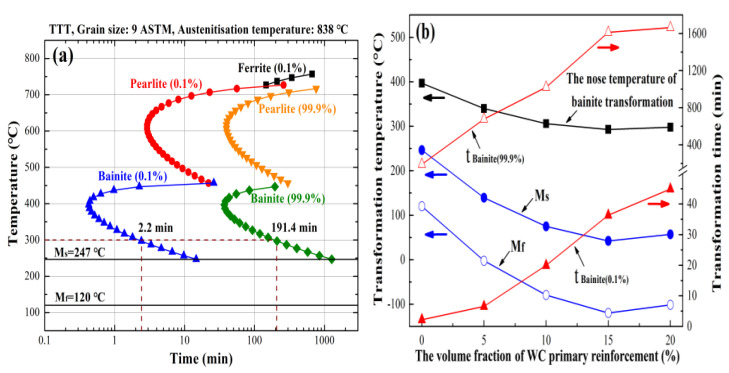
The austenite isothermal transformation kinetic of the composites: (**a**) time–temperature transformation (TTT) diagram of the bainite steel; (**b**) bainite and martensite transformation information of the composite with different volume fractions of WC primary reinforcement.

JMatPro software version 7.0 was applied to investigate the austenite isothermal transformation kinetics of the matrix of the composite using the general steel database (Figure 10). For the bainite steel (Figure 10a), the nose temperature of the pearlite transformation is 607 °C, with an incubation period of about 3 min; the nose temperature of the bainite transformation is 397 °C, with an incubation period of about 0.5 min. Meanwhile, the martensite transformation starts at 247 °C. Owing to the rapid cooling rate of the DLD process (10^3^~10^4^ °C/s), the pearlite transformation is completely prevented during the DLD process. In order to obtain the lower bainite microstructure, the transformation of martensite must be avoided the during cooling process and the following isothermal temperature has to be lower than the nose temperature of the bainite transformation. Hence, the preheating and isothermal temperature is set as 300 °C. According to the bainite transformation time at 300 °C, the isothermal treatment time was set to 200 min in this work.

The nose temperature of the bainite transformation and the martensite transformation temperature of the bainite steel matrix in the composite were also calculated using JMatPro software, as well as the bainite transformation time (Figure 10b). With the increased volume fraction of primary reinforcement in the composite, both the nose temperature of bainite transformation and the martensite transformation temperature decrease. Furthermore, both the incubation period and completion time of the bainite transformation increase significantly when the primary reinforcement content in the composite is increased. The lowest bainite transformation nose temperature of the composite matrix is about 300 °C, which ensures that the bainite structure obtained via isothermal treatment in the matrix is needle-like lower bainite. When WC primary reinforcement is added into the bainite steel matrix, the start temperature of martensite transformation (M_s_) in the matrix is still higher than room temperature, while the finish temperature of martensite transformation (M_f_) in the matrix is much lower than room temperature. Meanwhile, the time required for the matrix to complete bainite transformation is much longer than the isothermal treatment time (200 min) for the composites. As a result, a small part of the untransformed undercooled austenite transforms into high-carbon cryptocrystalline martensite, while most of the untransformed undercooled austenite maintains its original crystal structure, which leads to the formation of a large amount of RA structure in the matrix. Moreover, the content of RA increases with the increased solute W and C content in the bainite steel matrix, which has a proportional relationship with the volume fraction of primary reinforcement in the composites (Figure 2b).

### 4.3. Effect of the Adaptive Adjustment of the Matrix Microstructure on the Mechanical Properties of Composites

**Figure 11:**
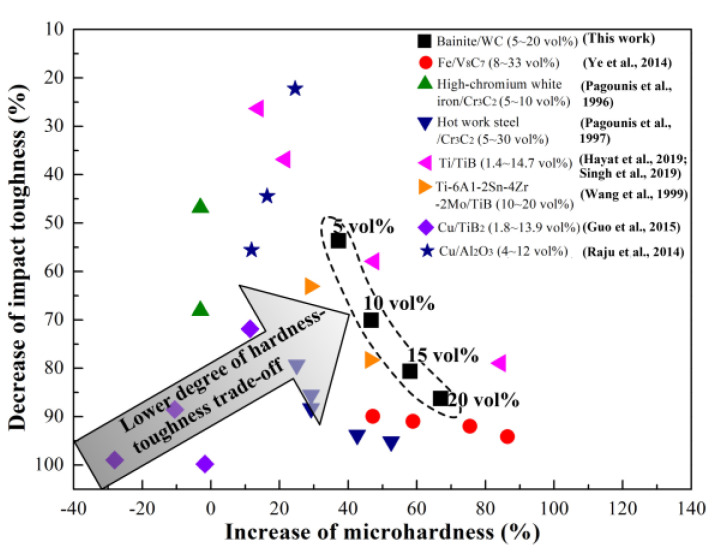
Hardness-toughness trade-off caused by the increased reinforcement volume fraction in MMCs (e.g., IMH = 100% × (HVBSC−20−HVBS)/HVBS, where IMH is the increase in microhardness, HVBSC−20 is the Vickers microhardness of the bainite steel matrix composite with 20 vol% primary reinforcement, and HVBS is the bainite steel) [52,53,54,55,56,57,58,59].

Since reinforcement particles in MMCs are usually hard and brittle, the increased volume fraction of the reinforcement particles results in an enhancement in the strength and hardness of MMCs and a reduction in the ductility and toughness of MMCs (i.e., hardness-toughness trade-off). Figure 11 presents the hardness-toughness trade-off caused by the reinforcement particles’ volume fraction in MMCs [52,53,54,55,56,57,58,59]. In order to facilitate a comparison between the mechanical properties of different MMCs, the microhardness and impact toughness are converted into the increase in microhardness and the decrease in impact toughness, respectively. Because the data point is close to the top right corner, the degree of hardness-toughness trade-off in the materials is low, and the hardness-toughness balance is good. The degree of hardness-toughness trade-off in the composite in this work is lower than that of most conventional MMCs, which can be attributed to the change in the matrix microstructure induced by the increased volume fraction of primary reinforcement. In conventional MMCs, the phase constituent and morphology of the matrix are almost not changed with the increased volume fraction of reinforcement, but the matrix microstructure of the composite in this work is altered with the increased primary reinforcement content. The residual austenite content in the steel matrix increases with the increased volume fraction of WC primary reinforcement, which is beneficial to improve the toughness of the steel matrix in composites [60]. The high volume fraction of reinforcement improves the hardness of the composite, while the adaptive adjustment of the matrix microstructure offsets a part of the reduction in impact toughness caused by the increase in the reinforcement volume fraction. As a result, the bainite steel matrix composite in this work demonstrates a better balance of hardness and impact toughness compared with most conventional MMCs.

## 5. Conclusions

The in situ bainite steel matrix composites with WC primary reinforcement were manufactured using direct laser deposition. The effect of the primary reinforcement volume fraction on the composite microstructure and its mechanical properties were investigated. With the increased primary reinforcement content, the adaptive adjustment of the matrix microstructure was obtained. Furthermore, the dependence of the adaptive adjustment of the matrix on the combination of hardness and impact toughness in the composites was evaluated. The main conclusions are as follows:

(1) The interaction of the primary composite powder irradiated by laser during DLD leads to significant changes in the phase constituent and morphology of the reinforcement and matrix of the composites at the same time. With the increased volume fraction of primary reinforcement, the main phase in the matrix changes from dominant α-Fe to a mixture of γ-Fe and α-Fe. Specifically, with the increased primary reinforcement content, the matrix microstructure is changed from lath-like bainite, granular bainite and few island-like retained austenite into needle-like lower bainite and plenty of block-like retained austenite, and the final reinforcement is changed from Fe_3_W_3_C into Fe_3_W_3_C and WC.

(2) The microhardness increases rapidly to 461 HV0.2 when the WC primary reinforcement volume fraction is 5 vol%, which is approximately 40% higher than that of the bainite steel. Moreover, the microhardness of composites with 20 vol% primary reinforcement is increased to 561 HV0.2. In contrast, the impact toughness of the bainite steel matrix composite decreases with the increased primary reinforcement volume fraction. However, the impact toughness of the composite when the primary reinforcement volume fraction is less than 10% is still higher than that of the U75V steel substrate, which can satisfy the demand of rail turnout service.

(3) Compared with the conventional metal matrix composites, the bainite steel matrix composites manufactured via DLD possess a much lower degree of hardness-toughness trade-off. The better combination of microhardness and impact toughness can be attributed to the adaptive adjustment of the matrix microstructure with the increased volume fraction of primary reinforcement, which provides new insights into obtaining new materials with a good combination of hardness and toughness.

## Figures and Tables

**Table 1:** Chemical composition (in wt%) of the Fe-based powder and U75V steel substrate.

	C	Si	Mn	Cr	Ni	Mo	Al	V	Fe
Substrate	0.78	0.66	0.96	/	/	/	/	0.05	Bal.
Powder	0.45	0.90	1.20	0.90	1.90	0.30	1.20	/	Bal.

## Data Availability

Research data are not shared.
